# Do we need to change the guideline values for determining low bone mineral density in athletes?

**DOI:** 10.1152/japplphysiol.00851.2021

**Published:** 2022-01-21

**Authors:** Kristin L. Jonvik, Monica K. Torstveit, Jorunn Sundgot-Borgen, Therese Fostervold Mathisen

**Affiliations:** ^1^Department of Physical Performance, Norwegian School of Sport Sciences, Oslo, Norway; ^2^Department of Sport Science and Physical Education, University of Agder, Kristiansand, Norway; ^3^Department of Sports Medicine, Norwegian School of Sport Sciences, Oslo, Norway; ^4^Faculty of Health, Welfare and Organisation, Østfold University College, Halden, Norway

**Keywords:** bone mineral density, guidelines, impact sports, reference, sport, *Z*-score

## INTRODUCTION

Bone mineral density (BMD) relates to bone strength, and low BMD is a risk factor for fractures and osteoporosis (1–3). In athletes, the nongenetic factor most commonly causing low BMD is prolonged and/or repeated periods with significant low energy availability (LEA), which also is associated with several serious, clinical impairments (4–6). These consequences of LEA in athletes range from short-term reductions in physical performance (e.g., due to low energy stores, impaired training adaptions and recovery, and increased injury risk), to long-term or even permanent illness or functional impairments (such as gastrointestinal dysfunction, impaired immune system, disturbance in hormonal function, and osteoporosis); a syndrome spectrum called relative energy deficiency in sport (RED-S) (5, 7). Interestingly, the reference scale for BMD evaluation is based on the normal population, and as such we may theoretically overlook athletes that already have reduced their BMD and are energy deprived. This potential masking of a RED-S-related symptom might be a result from interpreting BMD on wrong assumptions. Athletes, and specifically those representing high-impact sports and experiencing high mechanical loading, are expected to have higher BMD than age-matched nonathletes (8, 9). In fact, athletes from high-impact sports are expected to have a 5%–30% higher BMD compared with nonathletes (10, 11), and as such, we should expect that they have *Z*-scores above the population norm (≥0). Therefore, the American College of Sports Medicine (ACSM) and the International Olympic Committee (IOC) defines normal bone health in athletes from a *Z*-score ≥ −1.0, as opposed to ≥ −2.0 in the normal population (4, 5, 10). They propose that a *Z*-score < −1 warrants further follow-up and clinical examination of secondary risk factors. Athletes from low-impact sports, such as swimming and cycling, on the other hand, seem not to present with above normal levels of BMD (12–16). Based on current understanding on how high-impact loading positively affect BMD, a question to rise may be; is there a need for a sport-specific BMD reference values? And should such reference values depend on the type of sport/event, sex, and/or age (1)?

## WHAT DO WE KNOW?

Participation in high-impact sports with bone-specific loading activities increases BMD, both throughout adolescence (17) and adulthood (18). Such sports have weight-bearing endurance activities (e.g., tennis and running), activities that involve jumping (e.g., volleyball and basketball), and resistance exercise (e.g., weightlifting) (19). The differences appear to be exacerbated with continued training and competition beyond adolescence and is more pronounced in males than females (20). In low-impact sports, the lack of mechanical loading of the skeleton is an important factor contributing to BMD values similar to nonathletes (18).

Next to the mechanical loading of the sport, RED-S can strongly negatively impact bone health (7). This is particularly related to LEA caused by low energy intakes and/or high total energy expenditure and affected hormones regulating bone metabolism (21).

Short-term consequences of low BMD in athletes include increased risk of stress fractures and traumatic bone fractures (22). Extreme weight control behaviors, disordered eating, and eating disorders can lead to inadequate bone development and increase the risk for bone stress injuries (23). Markers of LEA are associated with a 4.5 times greater rate of bone injuries in national/world-class female and male distance athletes (24). On the other hand, participation in sports with multiaxial loading combined with having a regular menstruation during adolescence and young adulthood may reduce the risk of multiple bone stress injuries (25).

## WHAT DO WE NOT KNOW?

**Figure 1.**
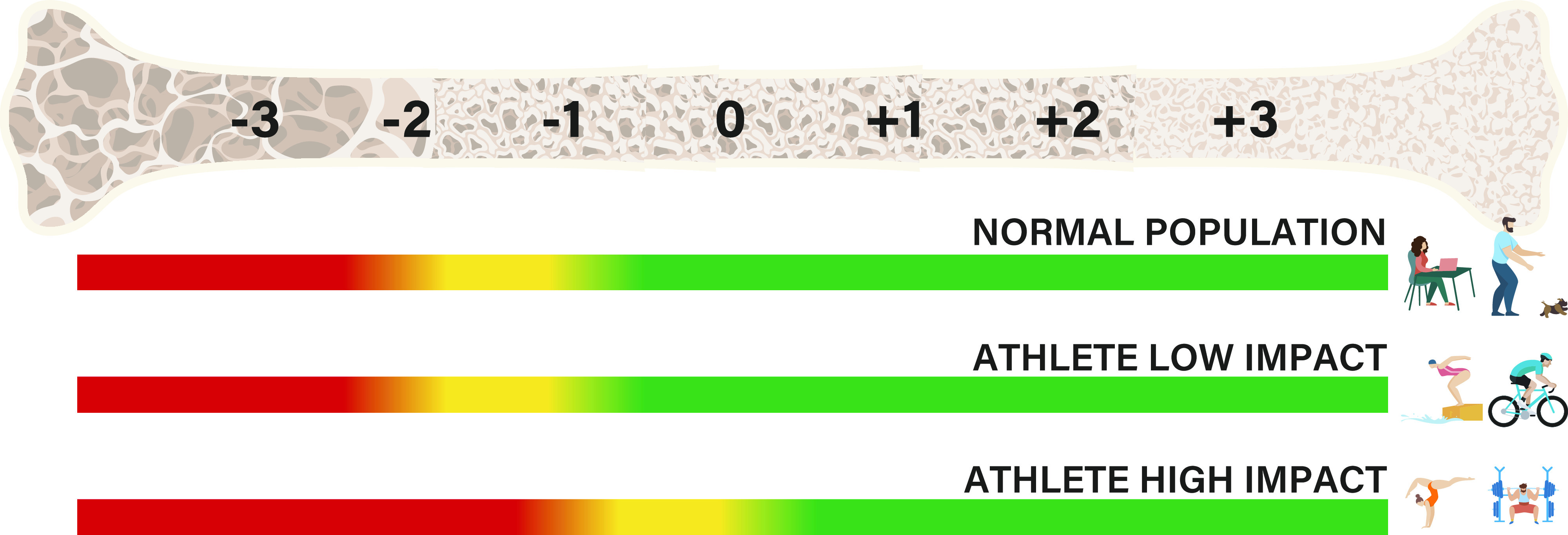
Proposed changes for the reference BMD *Z*-score range for the normal population and athletes participating in low impact and high impact sports. Graphic design: Thomas E. Fiskå, University of Agder, Norway. Used with permission. BMD, bone mineral density.

With no specified *Z*-score reference range for athletes, could we be ignoring important early sign information on impaired bone health? We do not know if athletes who are supposed to have a high *Z*-score due to the bone-specific loading of the sport, are overlooked if a *Z*-score between 0 and −1 is evaluated as normal, and not subjected to a follow-up or evaluation of secondary risk factors. A lack of athlete-specific normative data on BMD can result in the loss of opportunity to respond with early interventions directed to high-risk athletes, before more complex, clinical scenarios, such as bone stress injuries, occur. We propose the need for a sport/event-specific *Z*-score range, as suggested by the International Society for Clinical Densitometry (ISCD) (1), and as the example presented in Fig. 1. The yellow zone will in all cases imply further examination of secondary risk factors [e.g., disordered eating/eating disorders, reduced resting metabolic rate, low body fat percentage, or any of the other risk factors indicated by the IOC Risk Assessment Model (26)], and should be applied to high-impact sport athletes from a *Z*-score of 0 and below. This may improve the clinical evaluation of athletes to identify at risk athletes earlier, thereby providing an opportunity for optimal intervention and safer return to play for athletes developing impaired bone health.

There is a clear disadvantage by not having any sport/event-specific normative data for BMD, and additionally, there is a large knowledge gap with respect to bone microarchitecture. The latter is specifically important, as impaired bone microarchitecture, and as such impaired bone strength, could potentially be masked by an optimal BMD. On the other hand, despite a low BMD bone strength could be satisfactory if the bone architecture is good. Without knowledge about bone architecture, correct classification of high-risk group for fractures, impaired bone health, and further complications remains challenging.

To establish new cut-off ranges for athletes, there are several questions that needs to be answered: *1*) Is the suggested need for sport/event-specific *Z*-scores related to athletes at all ages? *2*) How many years of sport-specific loading is needed before sport-specific BMD occur? *3*) For how long do we see such sport-specific levels after cessation of the sporting career? *4*) Do we need to take sex-specific considerations, as the impact of training on bone health seems to be more pronounced in males than females (20)?

## WHAT DO WE NEED?

We suggest exercise physiology and sports medicine scientists unite to create a large, international sport/event-specific database of BMD. Data should additionally be sex- and age-specific, and include information on bone microarchitecture, years of exercise experience [years in total and in specific sport(s)] and controlled for factors that are known to effect BMD (e.g., family history of osteoporosis, menstrual history, medications, LEA, and ethnicity). There is a large diversity in BMD in both athletes and nonathletes that cannot be attributed solely to sex-, age- or physical activity-associated effects, indicating that other factors such as genetic variation also influence BMD. Heritability of BMD is estimated at 50%–85% (27), and numerous genes may play a role (28, 29). Taking genetics into account seems necessary, but definitely complicates the picture. As such, there will be a need for a very large number of athletes from different countries and ethnicities included in the database and we need to work together internationally to find the answers we are searching for.

We need more knowledge with respect to bone health of athletes from low impact sports. The cut-off ranges proposed in Fig. 1 might need to be further shifted for different sports/events and competitive level, but to conclude on this a substantial amount of data is needed. There is a long way to go to establish these data, and for now we need to always consider several factors when monitoring LEA and bone health. Based on the provided discussion, we cannot conclude that an athlete’s bone health is satisfactory based on a *Z*-score ≥ −1. An athlete’s *Z*-score should be interpreted based on knowledge about training history, type of sport/event, and changes over time.

## DISCLOSURES

No conflicts of interest, financial or otherwise, are declared by the authors.

## AUTHOR CONTRIBUTIONS

K.L.J. and T.F.M. conceived and designed research; K.L.J. and M.K.T. prepared figures; K.L.J. drafted manuscript; K.L.J., M.K.T., J.S.-B., and T.F.M. edited and revised manuscript; K.L.J., M.K.T., J.S.-B., and T.F.M. approved final version of manuscript.
